# Identification and expression characterization of the Phloem Protein 2 (*PP2*) genes in ramie (*Boehmeria nivea* L. Gaudich)

**DOI:** 10.1038/s41598-018-28953-2

**Published:** 2018-07-16

**Authors:** Pingan Guo, Yancheng Zheng, Dingxiang Peng, Lijun Liu, Lunjin Dai, Cong Chen, Bo Wang

**Affiliations:** MOA Key Laboratory of Crop Ecophysiology and Farming System in the Middle Reaches of the Yangtze River, College of Plant Science and Technology, Huazhong Agricultural University, 1 Shizishan Street, Hongshan District, Wuhan, 430070 Hubei Province China

## Abstract

Phloem protein 2 (PP2) is one of the most abundant and enigmatic proteins in sieve elements and companion cells, which play important roles in the maintenance of morphology, photoassimilate transportation and wound protection in higher plants, but to date, no *PP2* (*BnPP2*) genes had been identified in ramie. Here, a total of 15 full-length *BnPP2* genes were identified. These *BnPP2* genes exhibited different responses to abiotic stresses. Interestingly, the *BnPP2* genes are more sensitive to insect pests than to other stresses. A study of the *BnPP2-15* promoter revealed that pBnPP2-15 could drive specific GUS expression in the petiole, root and stamen and could also be induced by mechanical wounding and aphid infection in transgenic Arabidopsis lines. The subcellular localization of six BnPP2 proteins showed that GFP-BnPP2-1, GFP-BnPP2-6, GFP-BnPP2-7, GFP-BnPP2-9, GFP-BnPP2-11 and GFP-BnPP2-12 were predominantly located in the cytoplasm. These results provide useful information elucidating the functions of *BnPP2* genes in ramie.

## Introduction

Normal growth and development of land plants rely on the coordination of various tissues and organs, and the phloem plays a role as a bridge in this process. In vascular plants, the phloem tissue not only plays a necessary role in transporting photoassimilates and in long-distance delivery of macromolecules^1–3^ but also represents a central actor in organism coordination, such as the integration of various outside stimuli (mechanical injury, insect attack, fungal infection, etc.) to produce meaningful responses^4–10^. However, the mechanism of plant response to external stimuli via phloem remains unclear.

In angiosperms, the phloem mainly consists of sieve tubes, companion cells, sieve phloem fibers and phloem parenchyma cells. Mature sieve elements contain phloem-specific proteins (P-proteins), mitochondria, ER, and sieve element plastids^11^. P-proteins are a very complex component of the phloem tissue; so far, little is known about their function^12^. The soluble P-proteins and the structural P-proteins are two forms of P-proteins, both of which are found in phloem exudates^13–15^. The study of structural P-proteins has been more in-depth than that of soluble P-proteins. The structural P-proteins are divided into dispersive P-proteins and non-dispersive P-proteins depending on their chemical structure and function^12^.

Previous reports have shown that phloem exudates contain two predominant P-proteins (phloem protein 1 and phloem protein 2)^13,16^, which were the first dispersive P-proteins deciphered in detail with respect to structure and function at the molecular level^1,14,17^. Phloem filaments contain PP1 and PP2, which are linked by disulfide bridges. When plant phloem tissue is exposed to the air, the phloem filaments perform an anti-invasive function involving the oxidation of the phloem proteins cross-linked cysteine residues to form a sealed mechanical system^4,18^. Phloem protein 2 (PP2), a dimeric chitin-binding lectin^14,19^ and one of the most abundant and enigmatic proteins in the phloem sap^20^, plays important roles in the maintenance of morphology, photoassimilate transportation and wound protection in higher plants^1,3,21^.

As the phloem exudates of *Cucurbita* contain high concentrations of P-proteins, *Cucurbita* has been used as a model plant for studying P-proteins. As early as the end of the last century, an mRNA encoding the PP2 subunit of *Cucurbita maxima* had been cloned, and its specific localization in companion cells had been determined by *in situ* hybridization^14^. In addition, three genomic clones encoding PP2 were isolated from *Cucurbita maxima* in subsequent studies; meanwhile, they suggested that PP2 may be encoded by a small gene family (two to eight genes) by analyzing the copy number of *PP2* genes^17^. Wang *et al*.^22^ also obtained two cDNA clones encoding PP2 in *Cucurbita pepo*. The *PP2* genes were identified as one of the most abundant sequences in melon phloem sap, and at least five *PP2* genes were identified to be associated with cellular responses to hormones, stress and defense^23^. In celery (*Apium graveolens*), *AgPP2-1* and *AgPP2-2* are predominantly expressed in the phloem^20,24^. As a model plant, *Arabidopsis thaliana* was the first species in which all *PP2* genes were identified. In recent years, research into *AtPP2-A1* has become more active. *AtPP2-A1* and *AtPP2-A2* are two tandem genes, and they share very high amino acid homology with *CbmPP2*, *CmmLec26*, *AgPP2-1* and *AgPP2-2*; in addition, analysis of the promoter function of *AtPP2-A1* showed that it could drive dominant *GUS* expression in the phloem of tobacco and Arabidopsis^20^. Beneteau^18^ suggested that *AtPP2-A1* had different functions in the trafficking of endogenous proteins and in interactions with phloem-feeding insects; meanwhile, *AtPP2-A1*-overexpressing transgenic *Arabidopsis thaliana* had a deterrent effect on phloem-feeding activity^25^. Furthermore, *AtPP2-A1* was found to perform dual functions, both molecular chaperone activity and antifungal activity^26^.

Although the *PP2* of *Cucurbita* have been studied in detail at the protein level, and all *AtPP2* genes have been identified^20^, the expression patterns of the entire gene family under different stresses remain to be clarified. For vascular plants, the identification and expression pattern analysis of the *PP2* genes will help to elucidate the mechanisms of phloem response to external stresses.

Ramie (*Boehmeria nivea* L. Gaudich.), mainly grown in China, Philippines, Brazil, India and Viet Nam, is one of the most important phloem fiber crops in the world^27^. In view of the previous studies on the function of *PP2* genes, the *PP2* genes of ramie (*BnPP2*) may affect the yield and quality of phloem fiber, especially under stress conditions. Elucidation of the functions of the *BnPP2* genes may have positive impacts on the improvement of ramie resistance and on deciphering the mechanisms regulating response to adversity. However, no studies have yet characterized the *BnPP2* genes. In this study, fifteen *BnPP2* genes were identified, and we analyzed their phylogeny, gene structures, distribution of conserved domains and subcellular localizations. Meanwhile, we also investigated the expression patterns of all the *BnPP2* genes in various tissues or organs, as well as their expression profiles under biotic and abiotic stresses. In particular, we cloned and analyzed the promoter of *BnPP2-15*, which resembles *AtPP2-A1* (accession no. AT4G19840) very closely in its genetic evolution. This fundamental study will not only facilitate the further investigation of *BnPP2* genes with respect to their biological and molecular functions but also help us to understand the mechanism of phloem resistance, and provide a reference for the study of phloem protein 2 in other plants.

## Results

### Identification of *BnPP2* genes in transcriptome databases

The thirty AtPP2 protein sequences^20^ were used as queries for TBLASTN based on three transcriptome databases^28–30^. As a result, eighty-eight putative *PP2* genes were screened initially. After removing redundant sequences, we identified a total of fifteen *BnPP2* genes, which were named *BnPP2-1* to *BnPP2-15*, by using Clustal X. The lengths of the encoded proteins ranged from 177 amino acids to 441 amino acids, and the theoretical molecular masses range consistent with the amino acid lengths (20.09–47.3 kD) (Table 1).

**Table 1:** Summary of characterized *BnPP2* genes and proteins.

Name	DNA Sequence	ORF	Amino Acid	Molecular Mass	Intron Number
*BnPP2-1*	1974	897	298	33.53	2
*BnPP2-2*	2902	894	297	33.74	2
*BnPP2-3*	1743	855	284	31.82	2
*BnPP2-4*	1595	879	292	32.81	2
*BnPP2-5*	1401	843	280	31.79	2
*BnPP2-6*	1902	960	319	35.7	2
*BnPP2-7*	1621	789	262	29.84	2
*BnPP2-8*	1370	825	274	31.6	2
*BnPP2-9*	1808	816	271	30.69	2
*BnPP2-10*	3116	681	226	26.28	2
*BnPP2-11*	1054	897	298	33.6	2
*BnPP2-12*	1697	534	177	20.09	2
*BnPP2-13*	1176	1176	391	42.79	0
*BnPP2-14*	1326	1326	441	47.3	0
*BnPP2-15*	2346	708	235	27.13	2

### Structural organization, phylogenetic analysis and conserved domain identification of *BnPP2* genes

**Figure 1:**
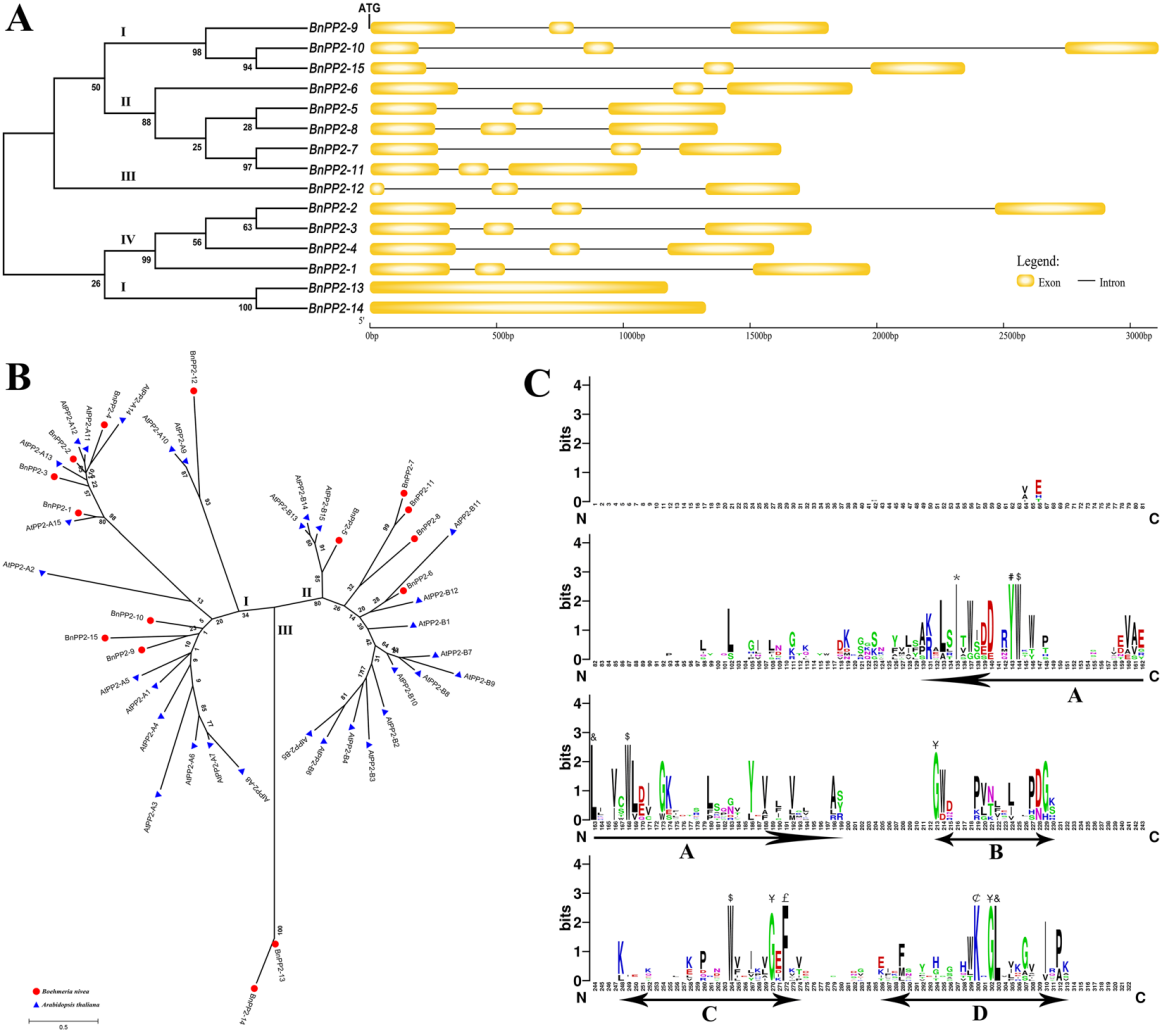
(**A**) Phylogenetic relationship of BnPP2 proteins and exon-intron structures of *BnPP2* genes. All sequences were left-justified by their initiation codons (ATG) and ended with termination codons (Not marked in the figure). (**B**) Phylogenetic relationship of PP2 proteins in *Boehmeria nivea* and *Arabidopsis thaliana*. The red solid circle indicates *Boehmeria nivea* and the blue solid triangle indicates *Arabidopsis thaliana*. Bar = 0.5, their length indicates the level of divergence among sequences. (**C**) Web-Logo analysis conserved domain of PP2 proteins. The letters of different colors indicated different amino acid abbreviations. The height of letter indicated that the position of the amino acid conservatism, the higher the more conservative. The conserved motifs A through D of the PP2 domain were underlined. *Conserved Ile residues. ^#^Conserved Tyr residues. ^$^Conserved Trp residues. ^&^Conserved Leu residues. ^¥^Conserved Gly residues. ^£^Conserved Phe residues. ^¢^Conserved Lys residues.

Analysis of the exon-intron structures of *BnPP2* genes can provide important information regarding the evolution of this gene family. The genomic sequences of *BnPP2* genes were cloned, and they ranged from 1054 bp to 3116 bp (Table 1). To obtain gene exon-intron structure information, the coding sequences were compared with the genomic sequences of all *BnPP2* genes. The *BnPP2* gene exon-intron structures are displayed in Fig. 1A. All the *BnPP2* genes contained two introns except *BnPP2-13* and *BnPP2*-*14*. To determine their evolutionary relationships and classify the BnPP2 proteins, a phylogenetic tree was constructed. The fifteen *BnPP2* genes were divided into four subgroups (I, II, III, IV and V) based on the results of the clustering analysis (Fig. 1A).

The phylogenetic relationships between *BnPP2* genes and other *PP2* genes with known functions from other species are useful for predicting their roles in ramie. In this study, thirty AtPP2 proteins were extracted from the Arabidopsis public gene database. Then, fifteen BnPP2 proteins and thirty AtPP2 proteins were used for the construction of the phylogenetic tree. All PP2 proteins of these two species were clustered into three subgroups (subgroup I, subgroup II and subgroup III) (Fig. 1B), and subgroup I contained eight BnPP2 proteins (BnPP2-1, BnPP2-2, BnPP2-3, BnPP2-4, BnPP2-9, BnPP2-10, BnPP2-15 and BnPP2-12). Five BnPP2 proteins (BnPP2-5, BnPP2-6, BnPP2-7, BnPP2-8 and BnPP2-11) were clustered into subgroup II. Only the two PP2 proteins of ramie were clustered into subgroup III. Obviously, these two proteins (BnPP2-13 and BnPP2-14) were the most distant from the other proteins (Fig. 1B).

The typical PP2 protein has a conserved PP2 domain signature, and previous studies have shown that the PP2 domain contains four conserved motifs (A, B, C and D)^20^. To examine the conservation of PP2 proteins between ramie and other species, we selected eight PP2 proteins from six different species (Winter squash, Cucumber, Melon, Celery, Arabidopsis and Ramie) for multiple sequence alignment. These PP2 proteins were CbmPP2-1 (accession number L31500), CmsLec26 (AF520581), CmmLec17 (AF520577), AgPP2-1 (AY114139), AtPP2-A1 (AT4G19840), BnPP2-2, BnPP2-5 and BnPP2-9. The result was presented by Web-Logo. The N-terminal extension of the PP2 proteins was poorly conserved, while the C-terminus contained four conserved motifs (A, B, C and D) (Fig. 1C). There were five highly conserved amino acid residues (Ile-135, Tyr-143, Trp-144, Leu-163 and Trp-168) in motif A, three (Trp-264, Gly-270 and Phe-272) in motif C, three (Lys-300, Gly-302 and Leu-303) in motif D and one (Gly-213) in motif B (Fig. 1C).

**Figure 2:**
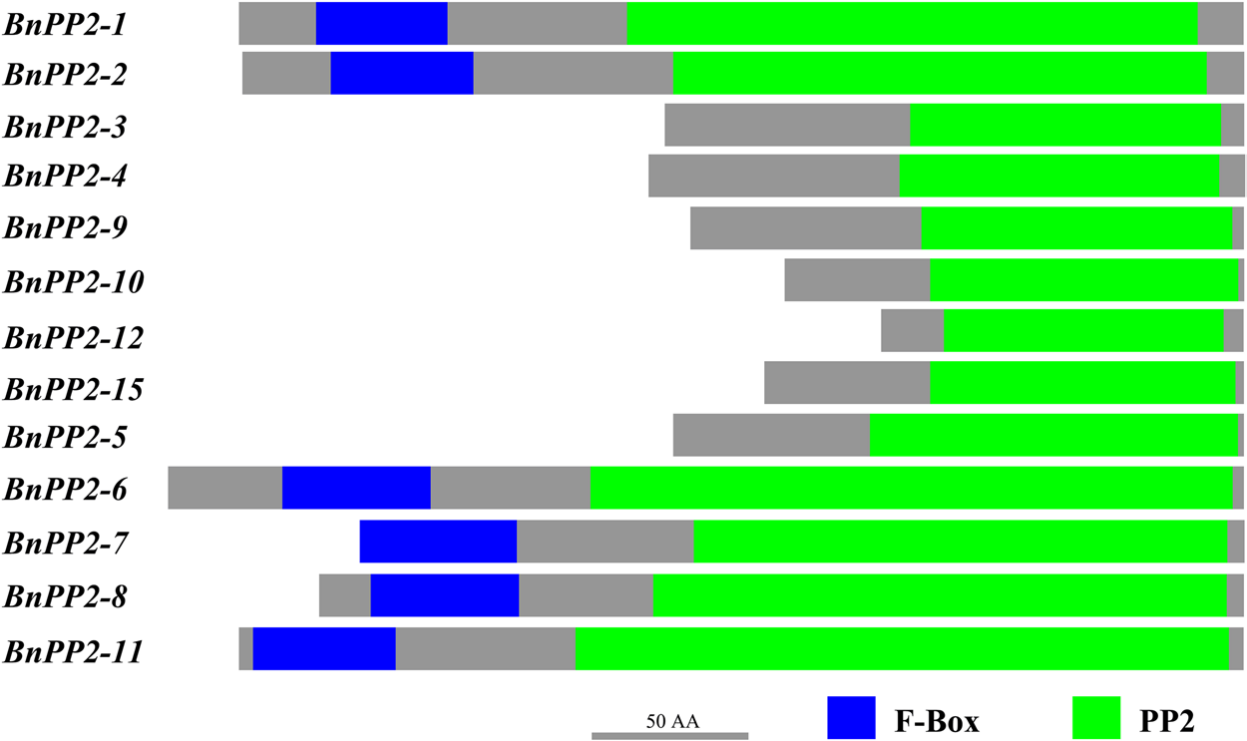
Schematic diagram of conserved domains in BnPP2 proteins. The green and blue blocks represented the PP2 domain and the F-BOX, respectively. Bar = 50 amino acids.

To more intuitively present the conserved domain of the BnPP2 proteins and their distribution, all BnPP2 amino acid sequences were analyzed using the Conserved Domain Architecture Retrieval Tool (CDART). Figure 2 shows that PP2 domain is predominantly present in BnPP2 proteins. The F-box, approximately 40 residues, is located in the N-terminal extension of six BnPP2 proteins (BnPP2-1, BnPP2-2, BnPP2-6, BnPP2-7, BnPP2-8 and BnPP2-11) (Fig. 2). Notably, two proteins (BnPP2-13 and BnPP2-14) were not predicted to have any conserved domains.

### Expression patterns of *BnPP2* genes in various tissues or organs

**Figure 3:**
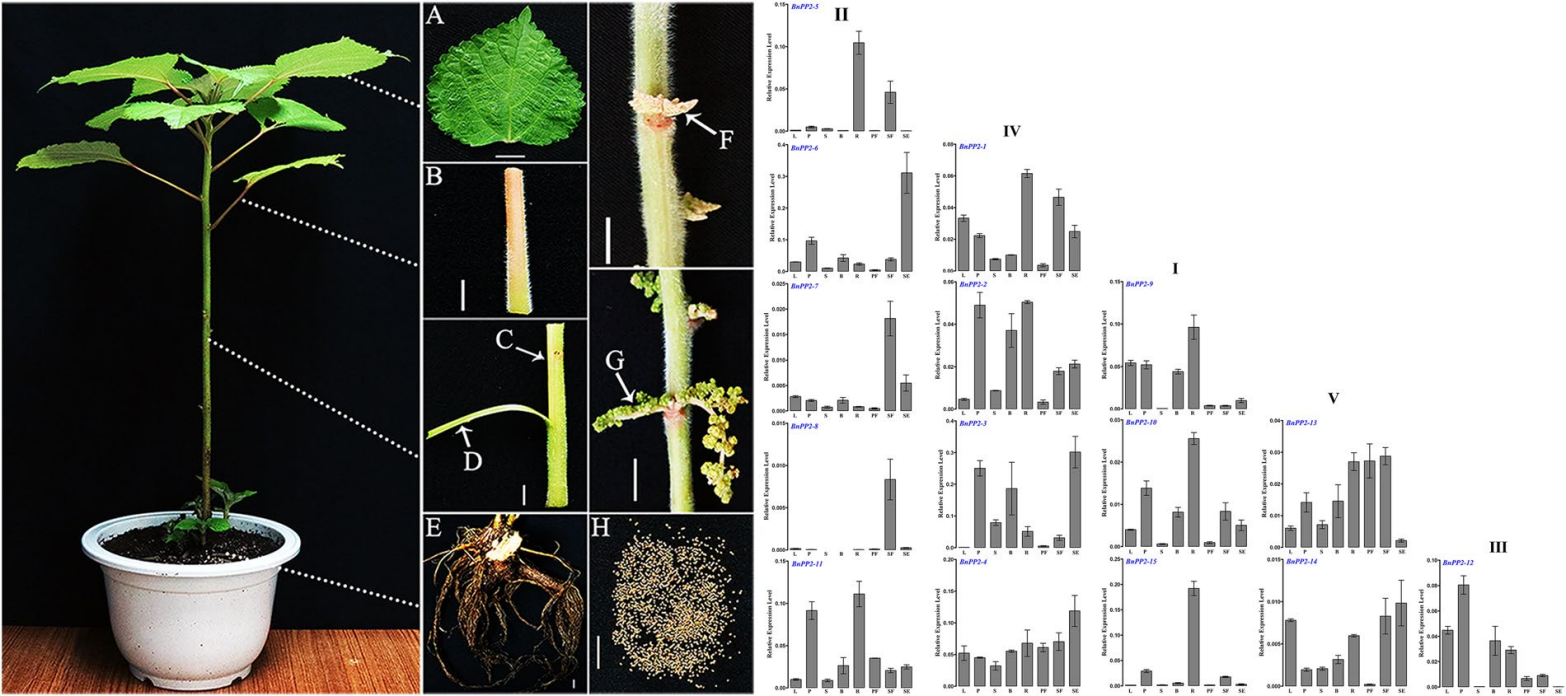
The diagram of eight different tissues or organs for RNA extraction in ramie (Left). (**A–H**) represented leaf (without main vein), petiole, stem, bark, root, pistillate flower, staminate flower and seed, respectively. C, D, F, and G were indicated by white arrows in this figure. Bar = 1 cm. Tissue-specific expression patterns of *BnPP2* genes (Right). The *BnPP2* genes were classified according to phylogenetic relationship (Fig. 1A). RNA was extracted from eight different tissues or organs of ramie and used for cDNA synthesis. L, leaf without main vein; P, petiole; S, stem; B, bark; R, root; PF, pistillate flower; SF, staminate flower; SE, seed. The data were presented as the mean ± SD of three separate measurements.

The relative expression levels of *BnPP2* genes in various tissues or organs were investigated by qRT-PCR using total RNA isolated from eight different tissues or organs (leaf without main vein, petiole, stem, bark, root, pistillate flower, staminate flower and seed) of ramie (Fig. 3). The results are shown in Fig. 3. The arrangement of histograms was based on the results of clustering in Fig. 1A. All the subgroup I members exhibited very low expression levels in the stem and exhibited the highest expression levels in the root. In subgroup II, all the genes had significant tissue expression biases, except *BnPP2-11*. *BnPP2-5* was expressed predominantly in the root and staminate flower, whereas *BnPP2-6* was strongest in the seed. *BnPP2-7* and *BnPP2-8* were expressed predominantly in the staminate flower. Furthermore, compared to those of the other genes, *BnPP2-8* had lower expression levels in all tissues or organs (Fig. 3). In subgroup III, *BnPP2-12* was highly expressed in the petiole and less expressed in the stem and seed. The subgroup IV members *BnPP2-1*, *BnPP2-2* and *BnPP2-4* were expressed in all tissues or organs (Fig. 3). However, *BnPP2-3*, another member of subgroup IV, was barely expressed in the leaf and pistillate flower compared to the other tissues. Meanwhile, in subgroup V, *BnPP2-13* was highly expressed in all tissues or organs except in the seed, whereas *BnPP2-14* had a lower expression level in the pistillate flower only (Fig. 3).

### Expression patterns of *BnPP2* genes under biotic and abiotic stresses

For the purpose of characterizing the functions of *BnPP2* genes under biotic and abiotic stresses, the expression patterns of *BnPP2* genes were investigated in response to abiotic stresses [Low-temperature (LT) (Fig. S1a), High-temperature (HT) (Fig. S1b), and Drought-stress (DS) (Fig. S1c) for the leaf without main vein, petiole, bark and stem; Mechanical-wounding (MW) for the leaf and bark (Fig. S2A1 and Fig. S2B1)] and biotic stresses [Insect-feeding (IF) (Fig. S2C and D) and Fungi- infection (FI) (Fig. S2F and G) for the leaf] by qRT-PCR.

**Figure 4:**
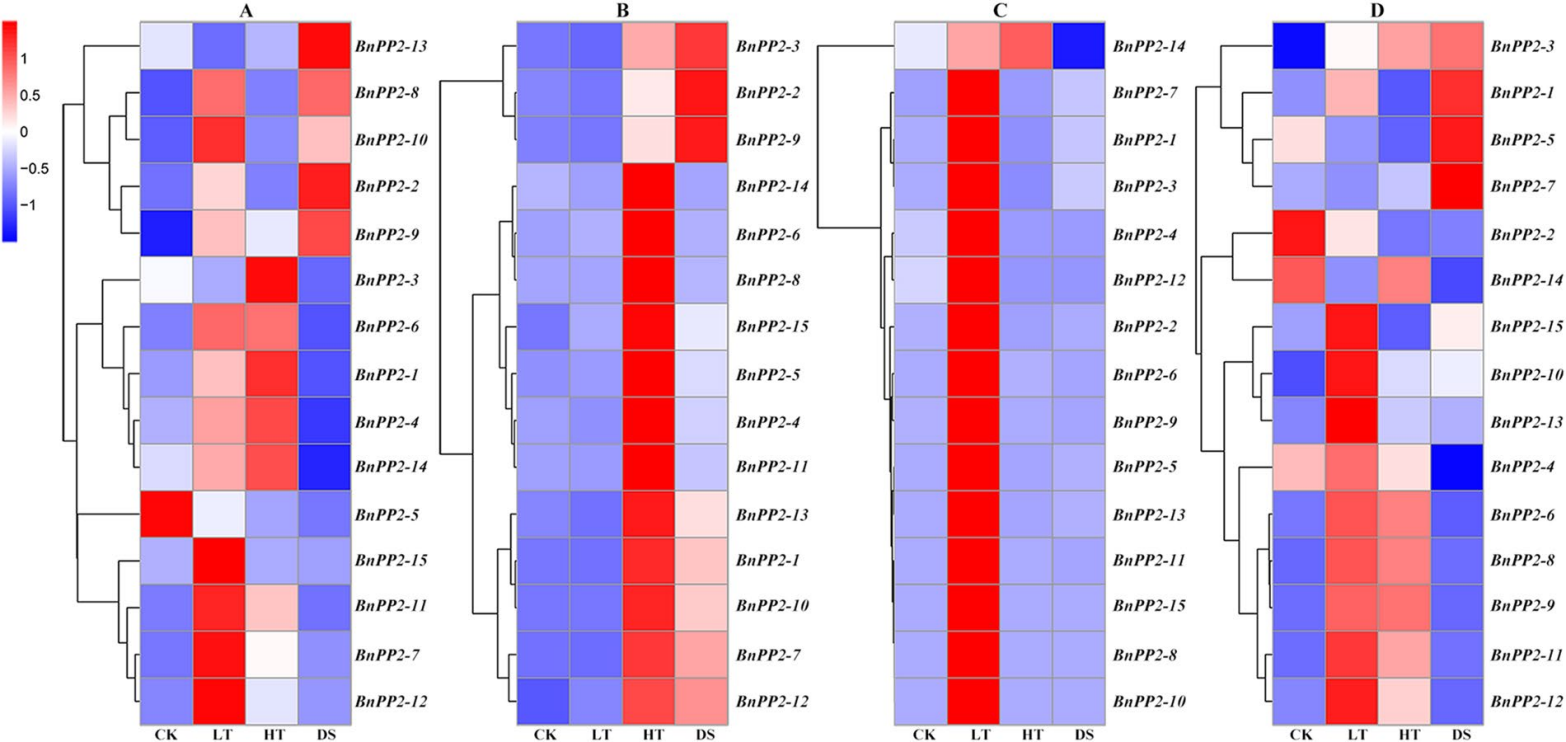
The relative expression level of *BnPP2* genes within four tissues in ramie under LT, HT and DS treatments. (**A–D**) indicates leaf (without main vein), petiole, bark and stem, respectively. The relative expression level (REL) was distinguished by the color of the gradient, the red for high expression level, while the blue for low expression level. The expression levels (using *eEF1α* as the endogenous control) were transformed (log^1+REL^) into colors in order to assess their overall expression levels. CK, plants from the normal condition for control; LT, low temperature; HT, high temperature; DS, drought stress. The color key in the upper left corner of the figure.

The relative expression levels of the *BnPP2* genes were evaluated in four different tissues (leaf without main vein, petiole, bark and stem) under different treatments (LT, HT and DS). The results are shown in Fig. 4. In leaves, compared to the control, the relative expressions levels of twelve *BnPP2* genes were up-regulated under the LT treatments while nine *BnPP2* genes were up-regulated under the HT treatments, and the relative expressions levels of five *BnPP2* genes (*BnPP2-2*, *BnPP2-8*, *BnPP2-9*, *BnPP2-10* and *BnPP2-13*) were also up-regulated under the DS treatment (Fig. 4A). However, the relative expression level of *BnPP2-5* was down-regulated in all three treatments (Fig. 4A). Figure 4B shows that LT treatment in the petiole had little effect on the expressions levels of *BnPP2* genes, whereas the HT treatment had a great impact, and DS treatment led to increases in eight genes’ relative expression levels. In contrast, the HT and DS treatments had little effect on the expression of *BnPP2* genes in the bark, but LT treatment had a great impact, except on *BnPP2-14* (Fig. 4C). The relative expression of *BnPP2-14* was down-regulated under DS treatment but up-regulated under LT and HT treatments in the bark (Fig. 4C). The relative expression of ten and seven *BnPP2* genes in stem was significantly up-regulated, respectively, under the LT and HT treatments, whereas the relative expression levels of just four genes (*BnPP2-1*, *BnPP2-3*, *BnPP2-5* and *BnPP2-7*) were significantly up-regulated under the DS treatment (Fig. 4D).

**Figure 5:**
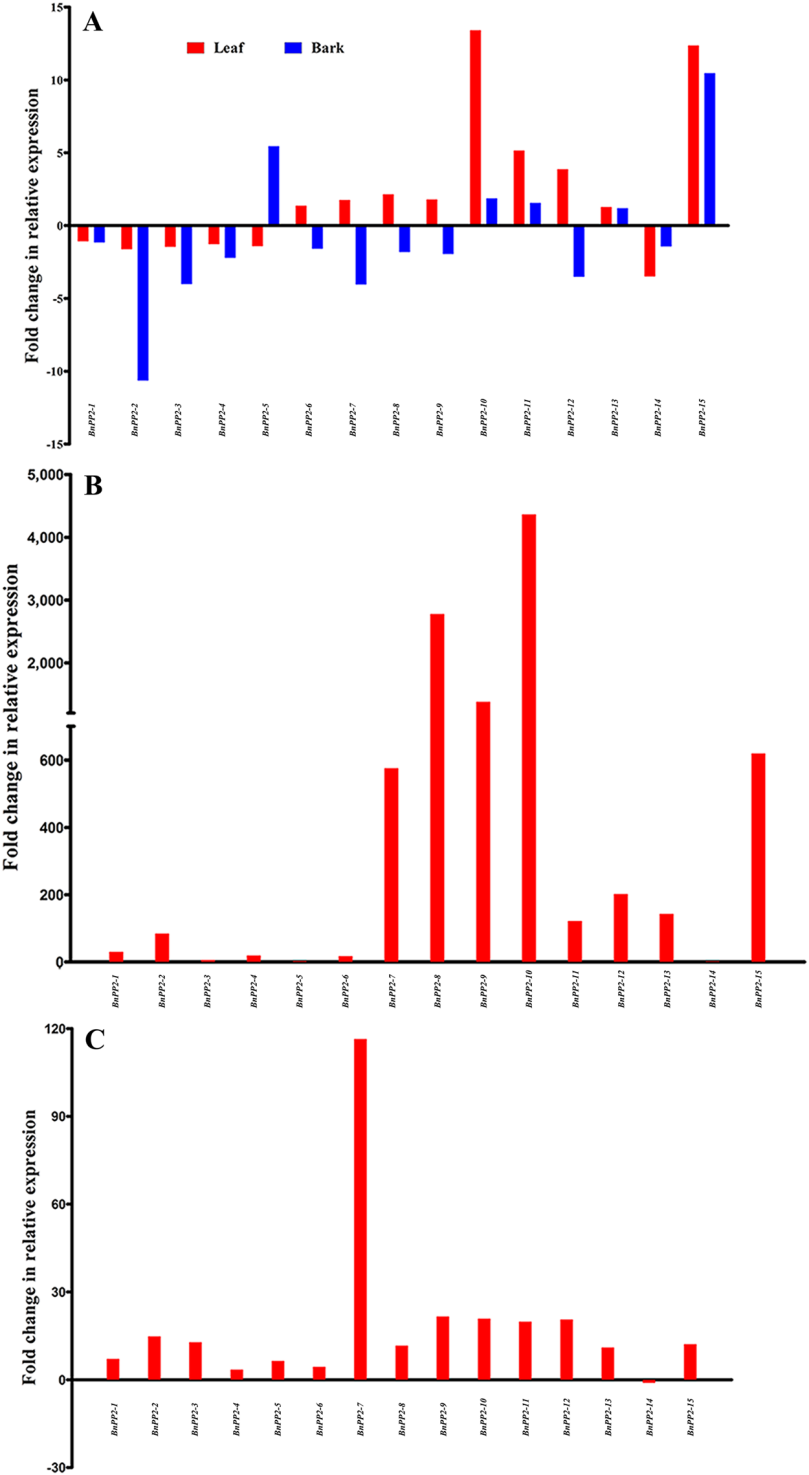
The fold change in relative expression of *BnPP2* genes in response to MW, IF and FI. Expression data were presented as fold change by comparing with the control. The blue color block represents data for leaf, while the red color block indicates bark. (**A**) MW (mechanical wounding); (**B**) Insect-feeding; (**C**) Fungal infection. The fold change was calculated by REL (Treatment)/REL (control) (up-regulated) or REL (control)/REL (Treatment) (down-regulated).

In the MW treatment, the expression patterns of nine *BnPP2* genes were similar in the leaves and bark. The expression levels of *BnPP2-1*, *BnPP2-2*, *BnPP2-3*, *BnPP2-4* and *BnPP2-14* were down-regulated, whereas the expression levels of *BnPP2-10*, *BnPP2-11*, *BnPP2-13* and *BnPP2-15* were up-regulated, and the expression levels of *BnPP2-6*, *BnPP2-7*, *BnPP2-8*, *BnPP2-*9 and *BnPP2-12* were up-regulated in leaves and down-regulated in the bark. In addition, the expression level of *BnPP2-5* was up-regulated in the bark and down-regulated in leaves (Fig. 5A).

There was a very significant up-regulation of *BnPP2* gene expression in leaves under the IF and FI treatments (Table S1). Expression of all the *BnPP2* genes was induced under IF treatment, especially that of the *BnPP2-8*, *BnPP2-9* and *BnPP2-10*, in which more than 1000-fold changes were induced compared to the control leaves (Fig. 5B). Although IF treatment also increased the relative expression levels of *BnPP2-3*, *BnPP2-5*, and *BnPP2-14*, they changed little compared to other *BnPP2* genes (Fig. 5B). In the FI treatment, the relative expression of all *BnPP2* genes was up-regulated, except that of *BnPP2-14*, but the fold changes in most of the relative expression levels were much lower than those in the IF treatment (Fig. 5C).

### Functional analysis of the promoter of *BnPP2-15* in transgenic *Arabidopsis*

**Figure 6:**
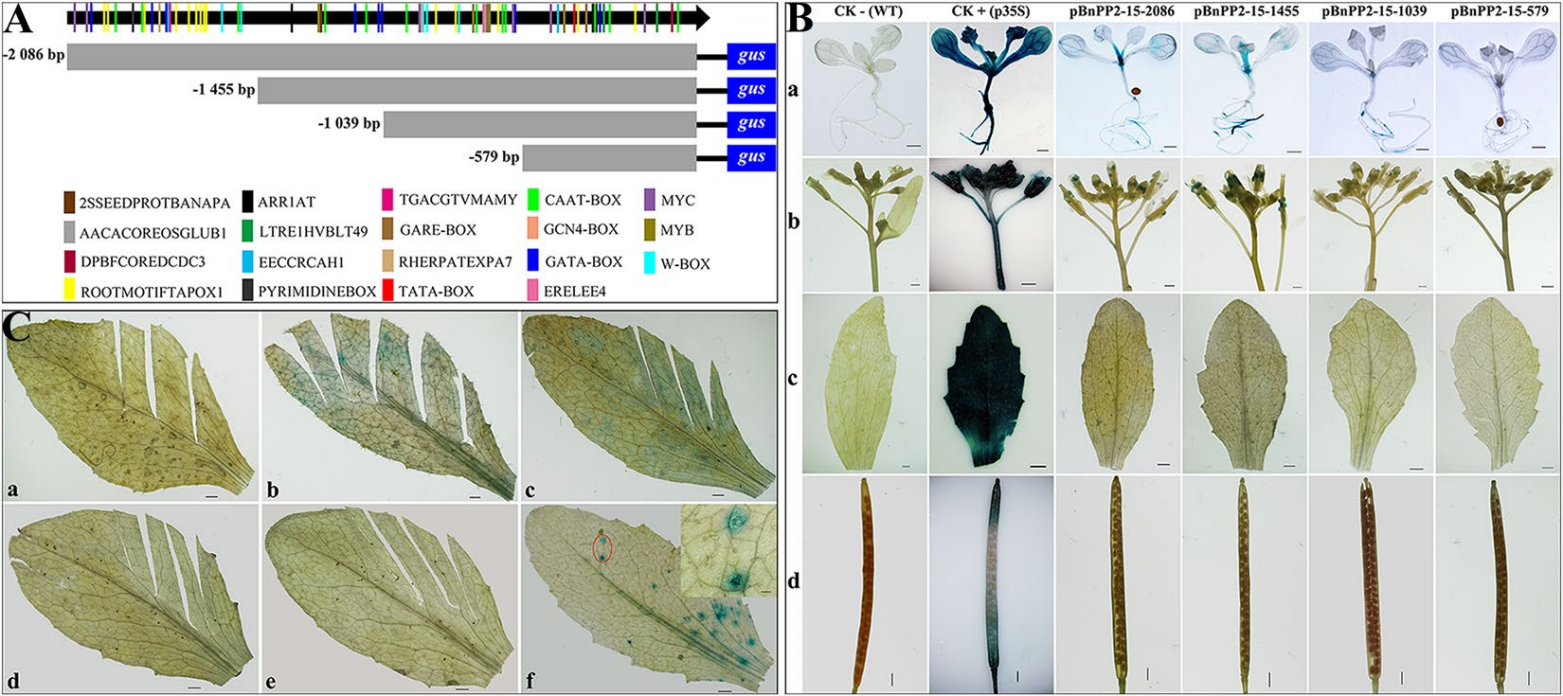
(**A**) Schematic diagram of truncated pBnPP2-15-2086 fused GUS constructs and distribution of *cis*-acting elements. The initiation codon was defined as +1. The different elements were indicated by different color patches. The position of the scissors indicates the location of the truncation of the promoter. The numbers on the left indicated the length of 5′- deletion fragments. (**B**) GUS histochemical assays in transgenic T3 Arabidopsis seedlings, flowers, mature leaves and siliques carrying pBnPP2-15-2086, pBnPP2-15-1455, pBnPP2-15-1039 and pBnPP2-15-579 constructs. CK−, wild-type; CK+, carrying CaMV 35S promoter constructs. a, b, c and d indicated seedlings, flowers, mature leaves and siliques, respectively. Bar = 0.1 mm. (**C**) GUS histochemical assays in mature leaves of transgenic T3 Arabidopsis under mechanical wounding and aphid infestation. a indicated leaf from wild-type for control; b, c, d and e indicated leaf from transgenic T3 Arabidopsis lines carrying pBnPP2-15-2086, pBnPP2-15-1455, pBnPP2-15-1039 and pBnPP2-15-579 constructs under mechanical wounding, respectively. f indicated transgenic T3 Arabidopsis lines carrying pBnPP2-15-2086 under aphid infestation. The red marker in Fig. 6Cf was enlarged in the upper right corner. Bar = 0.1 mm.

Phylogenetic analysis revealed that *BnPP2-15* is closely related to *AtPP2-A1* (Fig. 1B), and *AtPP2-A1* has been studied further. Thus, we selected the *BnPP2-15* and cloned its promoter. A 2086 bp 5′-flanking region fragment of *BnPP2-15* was isolated by using Universal Fast Walking (UFW). The promoter sequence is shown in Fig. S3 and was analyzed using the web tool-PLACE (Plant *cis*-acting regulatory DNA elements). Several putative *cis*-regulatory elements were deciphered in detail from the promoter sequence of *BnPP2-15* (Table S2). The distribution of key elements of the promoter was drawn, and serial 5′-flanking fragments were truncated based on the distribution of key elements in the promoter sequence (Fig. 6A).

GUS expression was detected in the petiole and root in 7-day-old transgenic T3 Arabidopsis seedlings carrying the pBnPP2-15-2086 construct (Fig. 6B). GUS expression also occurred in the stamens of the transgenic Arabidopsis, but almost no GUS activity was detected in the mature leaves and siliques (Fig. 6B). The results of GUS staining were consistent with the expression patterns of *BnPP2-15* in the different tissues or organs of ramie (shown in Fig. 3).

The 5′-deletion fragments were also fused to the *gus* reporter gene and transformed into Arabidopsis. GUS staining showed that the pBnPP2-15-1455 and pBnPP2-15-1039 constructs exhibited similar expression patterns to that of the pBnPP2-15-2086 construct (Fig. 6B). Furthermore, GUS staining was not visible in any organs of the transgenic *Arabidopsis* lines containing pBnPP2-15-579 (Fig. 6B).

To investigate the response of the *BnPP2-15* promoter to external injury, the mature leaves of transgenic Arabidopsis were scratched with scissors, and 30 min later, GUS staining was performed; in addition, transgenic Arabidopsis (pBnPP2-15-2086) leaves infected by aphids were used for GUS staining. GUS expression was detected in the wounded leaves of transgenic Arabidopsis lines containing pBnPP2-15-2086 and pBnPP2-15-1455 (Fig. 6Cb,Cc). GUS expression could not be detected in the wounded leaves of wild-type Arabidopsis or transgenic Arabidopsis lines containing pBnPP2-15-1039 and pBnPP2-15-579 (Fig. 6Ca,d,e). Moreover, GUS expression was induced at sites of aphid’ infestation (Fig. 6Cf).

### Subcellular localization of *BnPP2* genes

**Figure 7:**
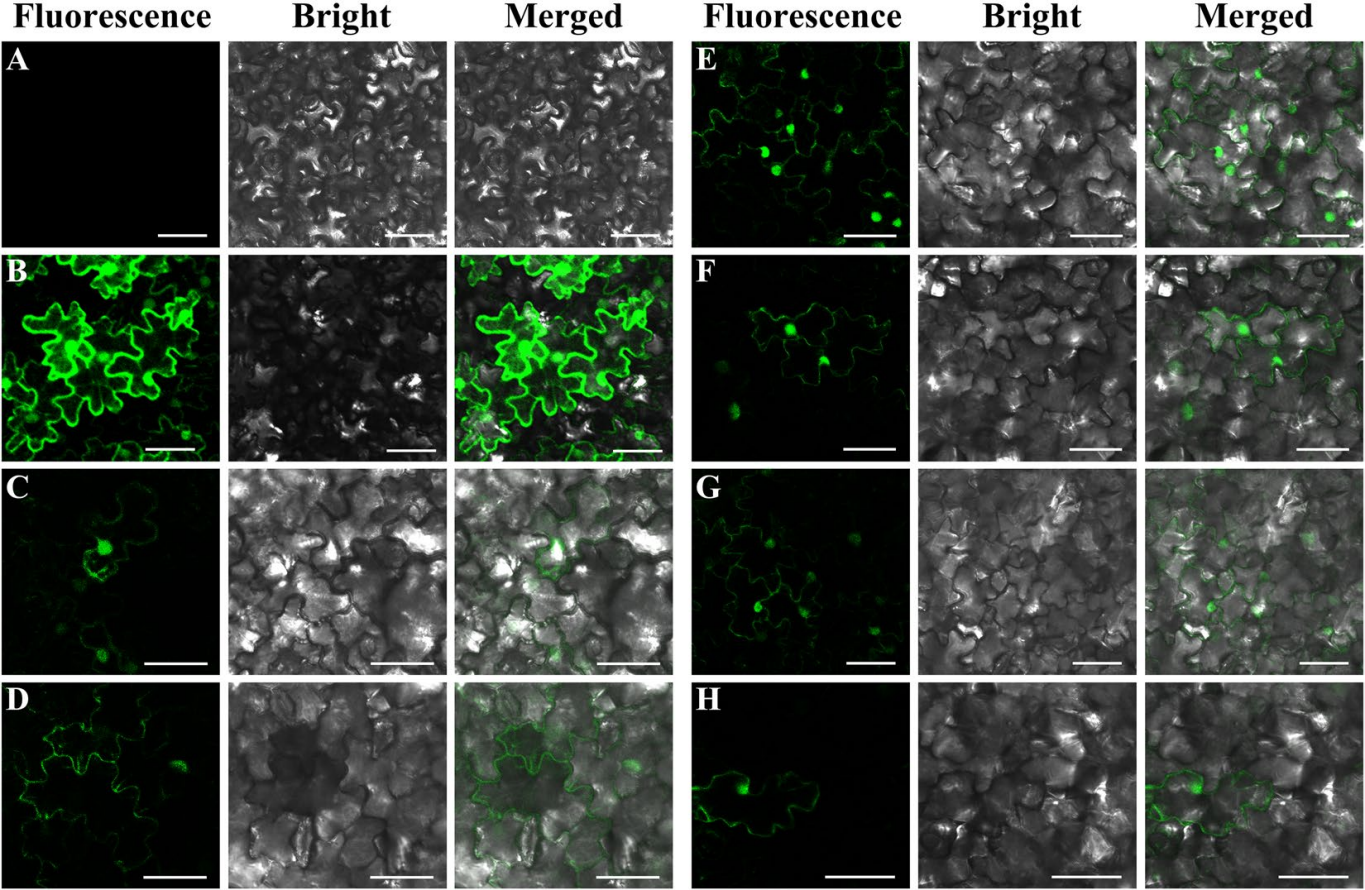
The sub-cellular localization of six GFP-BnPP2 proteins by transient expression in tobacco leaves. (**A**) Indicated tobacco cells that were not transiently transformed. (**B**) Indicated transiently transformed tobacco cells carrying pEGAD. (**C–H**) indicated GFP-BnPP2-1, GFP-BnPP2-6, GFP-BnPP2-7, GFP-BnPP2-9, GFP-BnPP2-11 and GFP-BnPP2-12, respectively. Bar = 50 µm.

To understand the role of BnPP2 proteins at the cellular level, it is helpful to decipher BnPP2 protein functions. We investigated the subcellular localization of all *BnPP2* proteins via transient transformation of *Nicotiana benthamiana* with green fluorescent protein (GFP) fused with each BnPP2 protein. Six of these fusions exhibited available results while the fluorescence signals of remaining fusions could not be effectively observed. The fluorescence signals of six GFP-BnPP2 fusion proteins (GFP-BnPP2-1, GFP-BnPP2-6, GFP-BnPP2-7, GFP-BnPP2-9, GFP-BnPP2-11 and GFP-BnPP2-12) were captured by the Laser Scanning Confocal Microscope. As shown in Fig. 7, confocal observation indicated that the fluorescence of six proteins was detected predominantly in the cytoplasm (Fig. 7C–H), while the fluorescent signal of GFP-BnPP2-1, GFP-BnPP2-7, GFP-BnPP2-9, GFP-BnPP2-11 and GFP-BnPP2-12 might also partly be localized to the nucleus, but this requires more accurate experiments to confirm.

## Discussion

The importance of phloem in vascular plants is self-evident. *PP2* may play an important role in assisting vascular plants against environmental stresses. In this study, fifteen *PP2* genes were identified in ramie, and the expression patterns of these genes in different tissues or organs were investigated as well as those in response to LT, HT, DS, MW, IF and FI. In addition, we cloned and analyzed the function of the *BnPP2-15* promoter; the subcellular localization of BnPP2 proteins in tobacco epidermal cells and the distribution of *BnPP2* mRNAs in the stem of ramie were also investigated.

In recent years, the study of related gene families has rapidly progressed with the prevalence of transcriptome sequencing. The molecular biological study of ramie has developed rapidly in recent years as well. Some gene families have been identified and characterized, such as *BnNAC* (NAC transcription factor) genes^31^, *BnCOL* (constans-like) genes^32^, *BnEXP* (expansin) genes^33^ and *BnSAUR* (small auxin-up RNA) genes^34^. However, the *PP2* genes have not been studied in ramie. Although *PP2* genes have been found in some angiosperms, the number of *PP2* genes present in the genome varies greatly among different species. Early studies using DNA blot analysis suggested that *PP2* was a small family in *Cucurbita maxima*, which contained two to eight genes^17^, whereas another study in the same period differed in this result; Wang *et al*. suggested that PP2 proteins were encoded by a gene family with a relatively large number of members (estimated as seven to fifteen per haploid genome) in *Cucurbita pepo*^22^. In addition, after the *Arabidopsis thaliana* genome sequence became available, a total of thirty *PP2* genes were identified in *Arabidopsis*^20^. In ramie, we identified fifteen *PP2* genes based on three transcriptome databases. Recently, a transcriptome study of the phloem and xylem in ramie was performed^35^. However, we did not find additional *PP2* genes based on their published data. Furthermore, we have reason to believe that all *BnPP2* genes will be identified for more comprehensive study with the completion of ramie genome sequencing.

The PP2 domain signature has become a key feature to identify *PP2* genes. Through CDART analysis, we found PP2 domains in thirteen BnPP2 proteins (see Fig. 2). The PP2 domain was located in the C-terminal extension of the PP2 protein. In previous studies, four conserved motifs (A, B, C and D) were found in the C-terminal extensions of PP2 proteins^20^, along with some amino acid residues that were extremely conserved in these motifs, such as the Trp residues in motif A and motif C (see Fig. 1C). The N-terminal extension of the PP2 protein was poorly conserved. This effect might be caused by the presence of different domains in the N-terminal extension. Three different domains are found in the N-terminal extension of AtPP2 proteins, including the TIR domain, which is reported in both animals and plants to be involved with immune receptors^36–38^. Another domain, the AIG1 domain, was believed to be associated with immune responses in plants^10,39^. The F-box domain is predominantly present in subgroup II AtPP2 proteins^20^. In plants, the F-box domain is well known to be involved in many regulatory processes including protein degradation, hormone and light signaling^40^. The presence of these domains indicates that PP2 proteins are involved in many important physiological and biochemical processes in plants. The F-box domain was also found in six BnPP2 proteins (Fig. 2). Although only the F-box domain was predicted in BnPP2 proteins, we speculated that there might be domains such as TIR and AIG1 among the unidentified BnPP2 proteins. In particular, no conserved domains were predicted in BnPP2-13 and BnPP2-14. *BnPP2-13* and *BnPP2-14* were the only two *BnPP2* genes that did not have introns, and they were distant from the other genes in genetic evolution (Fig. 1B). Their function remains to be further elucidated.

Previous studies have focused on the localization of *PP2* proteins and their mRNA at the cellular level. The tissue localization of the PP2 proteins in some species has been adequately studied, and many studies have shown that PP2 proteins are specifically expressed in the phloem^14,20^. In this study, we investigated the subcellular localization of six GFP-BnPP2 proteins in tobacco leaves. These results will provide a reference for future study of *BnPP2* gene’ functions. The difference from previous studies is that this study focused on the expression pattern of *BnPP2* genes at the transcriptional level. We evaluated the expression of fifteen *BnPP2* genes in eight tissues or organs by qRT-PCR, and the results showed that the expression patterns of *BnPP2* genes were significantly different, which suggested that *BnPP2* genes might be involved in a variety of physiological and biochemical processes. In addition, the relative expression of all *BnPP2* genes in the bark was always higher than that in the stem, except that of *BnPP2-5* (see Fig. 3). This result indicated that *BnPP2* genes tended towards expression in phloem rather than in xylem.

The transport of organic compounds in plants depends on the phloem and the transportation process is affected by changes in the external environment. Therefore, we evaluated the expression patterns of the *BnPP2* genes in response to external stresses. The *BnPP2* genes exhibited a complex expression pattern in response to abiotic stress. Notably, most of the *BnPP2* genes had a tissue preference for temperature reactions. The expression of most of these genes was significantly induced by high temperature in the petiole, while in the bark, it was significantly affected by low temperature (Fig. 4B,C). *BnPP2-8* was the gene with the lowest expression in various tissues or organs (Fig. 3), but it was strongly up-regulated in response to various stresses, showing a more than 5000-fold change in the petiole under the HT treatment (Table S1). It could be inferred that *BnPP2-8* might play an important role in response to stresses. The responses of *BnPP2* genes to mechanical wounding in leaves and bark were closely related to their genetic evolution. Most of the genes in subgroup I, subgroup IV and subgroup V showed consistent responses (simultaneously up-regulated or down-regulated in damaged leaves and bark) to external mechanical wounding, except that of *BnPP2-9*, whereas most of the genes in subgroup II and subgroup III showed perfectly opposite responses in damaged leaves and bark, except that of *BnPP2-11* (Fig. 5A). Although *BnPP2-13* and *BnPP2-14* had a close genetic relationship, there were inconsistencies in their expression patterns in the different parts of ramie and in their responses to stresses. *BnPP2-13* and *BnPP2-14* also showed completely opposite responses to mechanical wounding (Fig. 5A). In addition, the expression patterns of the *BnPP2* genes were surprisingly consistent in response to biotic stress. All the *BnPP2* genes exhibited up-regulation under the FI and IF treatments, except *BnPP2-14* (Fig. 5B,C). The results suggested that the susceptibility of *BnPP2* genes to biotic stresses was higher than that to abiotic stresses. It is worth considering that mechanical wounding and insect damage to the leaves are similar in physical performance, but the *BnPP2* genes showed dramatic differences in their responses to each treatment. The *BnPP2* genes were much more sensitive to insects than to mechanical wounding in this study.

Promoter function analysis has a positive effect on the elucidation of gene function. Figure 1B shows that *BnPP2-9*, *BnPP2-10* and *BnPP2-15* are more closely related to *AtPP2-A1* in genetic evolution. This result indicated that the functions of *BnPP2-9*, *BnPP2-10* and *BnPP2-15* may be similar to that of *AtPP2-A1*, which is one of the most thoroughly studied *AtPP2* genes^18,25,26^. However, the GUS staining revealed that the promoter of *BnPP2-15* exerts its function mainly in petioles, roots and stamens (Fig. 6B), while in contrast, the promoter of *AtPP2-A1* could drive GUS expression in the phloem of Arabidopsis and Citrus^20,41^. This result might indicate that these *PP2* genes (closely related in genetic evolution) are different in function. Furthermore, after scratching, GUS expression was induced in wounded leaves carrying the pBnPP2-15-2086 construct and pBnPP2-15-1455 construct (Fig. 6C). These results suggested that the position of the W-box affects the function of pBnPP2-15 in response to injury. Although the tissue-specific expression of *BnPP2-15* (Fig. 6B) differed from that of *AtPP2-A1*, their response to external lesions was consistent^18,25^.

In conclusion, we identified the *BnPP2* genes for the first time and conducted a more comprehensive investigation of them at the transcriptional level. Although the expression of different *BnPP2* genes varies widely in different tissues or organs, we still find that these genes are expressed preferentially in bark compared to stems, except *BnPP2-5*. In response to temperature stress, all genes showed regularity changes in the petiole and bark. At low temperatures, all genes were up-regulated in bark, and they were up-regulated in the petiole under HT conditions. The relative expression levels of most *BnPP2* genes could be significantly induced by insect-feeding, especially those of *BnPP2-7*, *BnPP2-8*, *BnPP2-9*, *BnPP2-10* and *BnPP2-15*. These results provide the basis for further study of whether these genes have insect-resistance potential. In this study, we successfully cloned a promoter using the UFW method for the first time. This experiment provides a new strategy for promoter cloning, especially for species without genomic data. Although promoters have been studied for many years, this study includes the first promoter cloned from ramie, which will provide a reference for the future cloning and analysis of ramie promoters.

## Methods

### Identification of *BnPP2* genes

To identify phloem protein 2 genes in ramie, *Arabidopsis* PP2 protein sequences were downloaded from TAIR (http://www.arabidopsis.org/) to use as the query to perform local TBLASTN searches against three pools of unigenes de novo assembled from the RNA-seq results of previous studies^28–30^. The principle of selecting candidate genes was based on the E-value cutoff, which was set as 1e^−10^. Subsequently, the obtained sequences were aligned based on the nucleotide sequence using Clustal X^42^. After alignment, the redundant sequences were removed, and the remaining sequences were used for further analysis. Open Reading Frame Finder (http://www.ncbi.nlm.nih.gov/projects/gorf/) and FGENESH (http://linux1.softberry.com/), the HMM-based gene structure predictor^43^ were used to confirm the initiation and termination codons for each gene. The theoretical molecular weight of each BnPP2 protein was determined via an online bioinformatics website (http://www.bioinformatics.org/sms/index.html).

### Gene structure analysis

Genomic DNA was extracted from the young leaves of ramie cultivar Huazhu No. 5 using an Omega Plant Genomic DNA Extraction Kit (Omega Bio-tek, CA, USA), according to the manufacturer’s protocol. The *BnPP2* genes were cloned based on polymerase chain reaction (PCR) by using ramie genomic DNA as the template. The *BnPP2* gene-specific primers are listed in Table S3. After confirmation by sequencing, we obtained the genomic DNA sequences of the *BnPP2* genes. The exon-intron structure of each *BnPP2* gene was displayed via Gene Structure Display Server 2.0 (http://gsds.cbi.pku.edu.cn/) by comparing the coding sequence and genomic sequence^44^.

### Conserved domain identification and phylogenetic analysis

The T-Coffee program was used to perform a multiple sequence alignment of the PP2 proteins from different species (http://www.ebi.ac.uk/Tools/msa/tcoffee/)^45^, and then we used Web-Logo (http://weblogo.berkeley.edu/logo.cgi) to represent the results^46^. The amino acid sequences were analyzed using the Conserved Domain Architecture Retrieval Tool (CDART)^47^. The multiple alignments of BnPP2 proteins were analyzed by the MAFFT algorithm^48^. Phylogenetic trees were constructed in MEGA 6.0 software with the default settings by using the maximum likelihood method with 1000 bootstrap replications^49^.

### Cloning and analysis of the *BnPP-15* promoter

The 5′-flanking region of *BnPP2-15* was isolated based on Universal Fast Walking (UFW)^33,50^. The promoter element and motif predictions were based on the online analysis tool PLACE (Plant *cis*-acting regulatory DNA element) (http://www.dna.affrc.go.jp/PLACE/)^51^.

### Plant materials, growth conditions and treatments

The experimental materials were Huazhu No.5, which was obtained from the ramie germplasm resources garden at Huazhong Agricultural University and planted in pots with a diameter of 25 cm. All materials were tested after incubation in the artificial climate chamber for 30 days under a 16 h light/8 h dark cycle at 25 °C light/22 °C dark (normal conditions).

For the LT and HT, plant samples were incubated in the artificial climate chamber until the emergence of the phenomenon of stress under a 16 h light/8 h dark cycle at 5 °C light/5 °C dark and at 42 °C light/42 °C dark, respectively. For the DS, plant material growth conditions were consistent with the normal conditions, except for water supply, until severe drought symptoms appeared. Then, the leaf (without main vein), petiole, bark and stem were harvested for each treatment (LT, HT and DS). For the MW^52^, we crushed the leaves and stem bark several times with a needle, which effectively wounded approximately 40% of the leaf and bark area. Plants were incubated for 90 min under normal conditions, and the leaves and stem bark were harvested subsequently. For the IF, the plant materials were used for feeding *Cocytodes coerulea* Guenee, which were captured from the ramie germplasm resources garden at Huazhong Agricultural University. The leaves were harvested after approximately 40% of the leaf area had been destroyed. For the FI, the a *Colletotrichum gloeosporioides* strain was isolated from ramie leaves and used to infect ramie leaves according to the method described by Zhang^53^. The leaves were harvested after obvious disease spots appeared on the infected ramie leaves. The corresponding untreated plant materials were used as the controls. All harvested samples were immediately immersed in liquid nitrogen and stored at −80 °C for further analysis. Each experiment was performed with three biological repeats.

### RNA isolation and quantitative real-time RT-PCR analysis

Total RNA from diverse tissues under different treatments was extracted with the RNAprep Pure Plant Kit (Tiangen Biotech, Beijing, China). Subsequently, the total RNA was reverse transcribed by TransScript One-Step gDNA Removal and cDNA Synthesis SuperMix (TransGen Biotech, Beijing, China), according to the manufacturer’s instructions.

The qRT-PCR was conducted with iTaq Universal SYBR Green Supermix (Bio-Rad, Hercules, CA, USA) on a Bio-Rad iQ5 Real-Time PCR System (Bio-Rad, CA, USA). The ramie elongation factor *eEF1α* was used as the endogenous control; this gene has been proven to be a stable internal reference gene based on preliminary experiments (data not shown). The sequences of the gene-specific primers used for qRT-PCR are listed in Table S4. The relative expression levels of the target genes compared with that of *eEF1α* were defined as ΔCt = (Ct _*Target*_ − Ct _*eEF1α*_). The relative expression level of each gene was calculated according to the 2^−ΔCt^ and 2^−ΔΔCt^ method^54^. Experiments were performed three times in triplicate. The data were analyzed statistically with SPSS 20.0 and visualized with the R programming language or GraphPad Prism 5.0.

### Expression vector construction and *Arabidopsis thaliana* genetic transformation

The promoter of *BnPP2-15* was truncated to form four segments according to the distribution density of its predicted *cis*-elements. Each of the four segments were introduced into the binary vector pBI121^55^ using a ClonExpress II One Step Cloning Kit (Vazyme Biotech, Nanjing, China). The relevant primers are listed in Table S5, and the T-DNA region of pBI121 is depicted in Fig. S4. The recombinant vectors were confirmed by sequencing, transferred into *Agrobacterium tumefaciens* GV3101, and then used to transform *Arabidopsis thaliana* using the floral dip method^56^.

### GUS activity detection

Seedling and organs from transgenic T3 *Arabidopsis thaliana* plants were analyzed by histochemical staining for GUS activity^57^. Samples were immersed in X-gluc reaction buffer at 37 °C for 3 to 12 h in a dark incubator. Then 90% ethanol and 70% ethanol were used for decolorization. GUS activity sites were observed by stereomicroscope (Olympus SZX16).

### Transient transformation and subcellular localization

The full-length *BnPP2* cDNAs were obtained via PCR. The PCR products were cloned into the binary vector pEGAD^58^ using the ClonExpress II One Step Cloning Kit. The relevant primers are listed in Table S5, and the T-DNA region of pEGAD is depicted in Fig. S4. The recombinant vectors were confirmed by sequencing and transferred into *Agrobacterium tumefaciens* GV3101. *Agrobacterium tumefaciens* containing the recombinant plasmid was injected into the leaf epidermis of *Nicotiana benthamiana*, which had been incubated in the greenhouse for 30 days at 25 °C in 14 h light and 10 h dark^59^. After injection, the tobaccos plants were replaced in the greenhouse for 48 h. Next, the inoculated leaves were separated for fluorescence microscopy by using the laser scanning confocal microscope (Olympus FV1200).

### Availability of supporting data

The nucleotide sequence reported in this paper has been submitted to NCBI with accession numbers [(*BnPP2-1*) MF362959, (*BnPP2-2*) MF362960, (*BnPP2-3*) MF362961, (*BnPP2-4*) MF362962, (*BnPP2-5*) MF362963, (*BnPP2-6*) MF362964, (*BnPP2-7*) MF362965, (*BnPP2-8*) MF362966, (*BnPP2-9*) MF362967, (*BnPP2-10*) MF362968, (*BnPP2-11*) MF362969, (*BnPP2-12*) MF362970, (*BnPP2-13*) MF362971, (*BnPP2-14*) MF362972 and (*BnPP2-15*) MF362973].

## Electronic supplementary material

Supplementary Information
