# Supplementary material for: Identification and expression characterization of the Phloem Protein 2 (PP2) genes in ramie (Boehmeria nivea L. Gaudich)
Source: Sci Rep. 2018 Jul 16;8:10734. doi: 10.1038/s41598-018-28953-2 (PMC6048116; doi:10.1038/s41598-018-28953-2)
Supplement: Supplementary file 1 — Supplementary Information [file 41598_2018_28953_MOESM1_ESM.pdf]

Supplementary Information

Title

Identification and expression characterization of the Phloem Protein 2 (*PP2*) genes in ramie (*Boehmeria nivea* L. Gaudich)

Authors

Pingan Guo[1]

xc-guo12@hotmail.com

Yancheng Zheng[1]

870428051@qq.com

Dingxiang Peng[1]

pdxiang@mail.hzau.edu.cn

Lijun Liu[1]

liulijun@mail.hzau.edu.cn

Lunjin Dai[1]

dailunjin@gmail.com

Cong Chen[1]

chencong1992@webmail.hzau.edu.cn

Bo Wang[1*]

wangbo@mail.hzau.edu.cn

[1]MOA Key Laboratory of Crop Ecophysiology and Farming System in the Middle Reaches of the Yangtze River, College of Plant Science and Technology, Huazhong Agricultural University, #1 Shizishan Street, Hongshan District, Wuhan 430070, Hubei Province, China, Tel/fax: +86 27 87287136.

[*]Corresponding author

Tab. S1. The fold change of the relative expression level of *BnPP2* genes under different treatment. Fold change = REL (Treatment) / REL (Control) (REL: relative expression level; LT: low temperature; HT: high temperature; DS: drought stress; MW: mechanical wounding treatment; IF: insect feeding; FI: fungal infection).

Gene	Leaf (without main vein)						Petiole			Bark				Stem		
	LT	HT	DS	MW	IF	FI	LT	HT	DS	LT	HT	DS	MW	LT	HT	DS
BnPP2-1	2.07	3.08	0.52	0.92	30.42	7.11	0.81	18.15	10.01	9.42	0.39	1.63	0.86	1.65	0.80	2.12
BnPP2-2	1.80	1.06	2.61	0.62	84.45	14.85	0.57	7.38	18.65	12.97	0.41	0.84	0.09	0.61	0.30	0.32
BnPP2-3	0.68	2.05	0.41	0.68	5.79	12.83	0.42	10.65	16.14	6.38	0.52	1.42	0.25	1.66	1.90	2.02
BnPP2-4	2.11	2.69	0.28	0.78	19.28	3.45	0.60	10.69	2.03	6.89	0.28	0.27	0.45	1.08	0.96	0.68
BnPP2-5	0.33	0.16	0.06	0.70	3.00	6.43	2.01	64.64	9.27	212.32	0.06	2.85	5.45	0.43	0.24	1.78
BnPP2-6	4.64	4.48	0.43	1.37	17.44	4.40	2.73	61.12	2.86	29.70	1.53	0.56	0.63	7.81	6.77	0.41
BnPP2-7	14.80	6.16	1.85	1.76	576.45	116.46	0.89	32.11	21.63	51.75	0.13	5.99	0.25	0.56	1.36	6.45
BnPP2-8	3.22	1.33	3.26	2.15	2778.25	11.69	4.21	5763.35	260.24	70.97	0.98	1.32	0.55	116.32	100.62	3.03
BnPP2-9	2.86	2.29	3.67	1.79	1381.24	21.59	0.39	11.01	26.82	27.59	0.57	0.12	0.51	48.46	46.05	0.65
BnPP2-10	11.81	2.37	7.61	13.41	4364.11	20.87	0.93	38.24	19.13	275.00	1.02	1.56	1.87	4.33	2.13	2.29
BnPP2-11	3.05	2.10	0.93	5.16	121.77	19.83	0.50	48.87	3.88	131.53	1.57	0.64	1.56	5.98	4.36	1.07
BnPP2-12	7.66	2.54	1.23	3.87	202.26	20.54	3.26	21.11	16.20	6.85	0.07	0.09	0.28	11.83	6.21	0.04
BnPP2-13	0.51	0.81	2.12	1.27	143.00	11.04	0.20	17.01	7.55	30.63	0.51	1.14	1.19	3.72	1.46	1.29
BnPP2-14	1.60	2.05	0.12	0.29	2.57	0.91	0.50	10.63	0.65	1.40	1.65	0.26	0.70	0.42	0.92	0.26
BnPP2-15	10.91	0.84	0.46	12.37	619.60	12.19	5.60	46.48	11.86	1378.64	1.32	3.09	10.47	4.04	0.39	2.03

Table S2. Putative cis-acting elements in the BnPP2-15-2086 by PLACE.

Name of *cis*-element	Sequence	Position from translation start site	Function
2SSEEDPROTBANAPA	CAAACAC	-1247	Conserved in many storage-protein gene promoters
AACACOREOSGLUB1	AACAAAC	-701	Involved in controlling the endosperm-specific expression
ABRE-BOX	ACGTG	-82	Early responsive to dehydration
ACGTATERD1	ACGT	-1980 -1783 -1744 -1403 -121	Induction by dehydration stress and dark-induced senescence
ARR1AT	NGATT	-1342 -345 -334	Transcriptional activators
BIHD1	TGTCA	-1991 -1845 -1436 -809 -597 -255 -153	Involved in disease resistance responses
CAATBOX	CAAT	-1925 -1838 -1519 -1232 -1096 -961 -732 -645 -636 -340 -291 -64	Sequences responsible for the tissue specific promoter activity
CACTFTPPCA1	YACT	-1900 -1860 -1697 -1578 -1486 -1270 -1013 -1003 -965 -887 -794 -783 -654 -539 -536 -505 -486 -466 -372 -260	*Cis*-Regulatory elements for mesophyll-specific gene expression
CGACGOSAMY3	CGACG	-832	May function as a coupling element for the G box element
CPBCSPOR	TATTAG	-1075 -476	Cytokinin-enhanced Protein Binding site
CURECORECR	GTAC	-1237 -261 -163 -25	Copper- and oxygen–responsive elements
DPBFCOREDCDC3	ACACNNG	-84	ABA-responsive and embryo-specification element
EBOXBNNAPA	CANNTG	-2062 -2019 -918 -610 -486 -174	*Cis*-element binding BHLH factor involved in light responsiveness and tissue-specific activation
EECCRCAH1	GANTTNC	-1753 -461	Consensus motif of the two enhancer elements
ERELEE4	AWTTCAAA	-707	Ethylene responsive element
GARE-BOX	TAACARR	-694 -367	Gibberellin-responsive element
GATA-BOX	GATA	-2004 -1805 -1758 -1133 -1054 -1045 -605 -322	Required for high level, light regulated, and tissue specific expression
GCN4-BOX	TGAGTCA	-914	Required for endosperm-specific expression
GT1-BOX	GRWAAW	-1173 -1154 -939 -722 -97	GT-1 binding site in many light-regulated genes
GTGANTG10	GTGA	-1391 -76	Cis-regulatory elements for late pollen gene
INRNTPSADB	YTCANTYY	-1998 -1917 -1896 -820 -656 -342	Light-responsive transcription
LTRE1HVBLT49	CCGAAA	-311 -133	Low-temperature-responsive element
MYB1AT	WAACCA	-741	MYB recognition site
MYBGAHV	TAACAAA	-694	Central element of gibberellin (GA) response complex
MYBPLANT	MACCWAMC	-1254	Plant MYB binding site
MYBST1	GGATA	-439	Core motif of MybSt1 binding site
MYCCONSENSUSAT	CANNTG	-2062 -2019 -918 -610 -486 -174	MYC recognition site
NODCON2GM/ OSE2ROOTNODULE	CTCTT	-650	Nodule specificity of cis-acting regulatory elements
NTBBF1ARROLB	ACTTTA	-1859	Required for tissue-specific expression and auxin induction
POLLEN1LELAT52	AGAAA	-1174 -933 -363	Responsible for pollen specific activation

PROLAMINBOX	TGCAAAG	-1280	Minimal cis-element requirements for endosperm specific gene expression
PYRIMIDINEBOX	CCTTTT	-1871	Gibberellin-respons cis-element of GARE & involved in sugar repression
RHERPATEXPA7	KCACGW	-1785	Root Hair-specific cis-Elements
ROOTMOTIFTAPOX1	ATATT	-1964 -1950 -1829 -1724 -1683 -1660 -1644 -1633 -1624 -865 -799 -659 -395 -205	Root specificity element
SREATMSD	TTATCC	-1591	Sugar-repressive element
TATA-BOX	TATA	-405	Essential for promoter recognition
TATCCAOSAMY	TATCCA	-1590	Element Controls Tissue-Specific Sugar Sensitivity
TBOXATGAPB	ACTTTG	-553	Light-activated transcription;
TGACGTVMAMY	TGACGT	-1746	Cis-Element for Expression in Cotyledons of Germinated Seeds
W-BOX	TGACY	-1574 -787 -1512 -907 -896	Elicitor-induced activation of transcription via W box-related

* N indicates A, C, G or T; W indicates A or T; Y indicates C or T; R indicates A or G.

Table S3. The sequence of *BnPP2* gene-specific primers

Name	Sequence (5’-3’)
BnPP2-1	CACTCCACAATCCCATTCCTCT
	TGTGCATGGTGTTATGATCTTCC
BnPP2-2	GGTTCAAATTTTATGGGGACAAT
	TCATCTTAATAATAGGTTTTTCCT
BnPP2-3	CCTGAGTGCTAGTTAAATCACCC
	CCACCAGAACTCAAATCTCTTCC
BnPP2-4	GATCGTCTGTCTCTATGGGTTC
	ATAATGACAAATCTTCCTACGTGG
BnPP2-5	ATGTTACCAGAAGACTGTGTCTCA
	TGACACTTTCTTGTTTGGTGGTTAG
BnPP2-6	CACACAAATCCCCTTCTGCATTC
	TTGATTACATGGTTTCCTCGGCA
BnPP2-7	AGAGAGCTCAACAATGGAGATGA
	CTTGAAATGATTGCGACCCTTACA
BnPP2-8	ATGGAAGAAAAGGGACTAATGGACT
	TCATGCAATCTCCTTGGGGCG
BnPP2-9	GTTTATGTAAATAAATTAGAATATG
	CTAATCTTTTGCTGGCTGTATT
BnPP2-10	CCGGAATTAGTTGGCGACGAAA
	GATTATTCGCCATCCGTTTACAC
BnPP2-11	AAAGAGAGCTCAACAATGGAGATGA
	CAAACAGGGAAACAGTGCCAAG
BnPP2-12	GGAGAGAGTTTGAGAGAAGCCC
	CGATTAACTTGCACGATCACCAT
BnPP2-13	AATTCCCTTCAGCGTTTATAGGC
	CACACTAAGACCTTCCACAAATCAC
BnPP2-14	TCGATGGACTTGCAATTGGTG
	GTGGACTGAGATGAGGCTTGT
BnPP2-15	AGCGTCTCGCGAGTACAAGCC
	TCCCAAGCAATTTCTCAATCCC

Table S4. The sequence of gene-specific primers used for qRT-PCR

Name	Sequence (5'-3')	Length of product (bp)
qBnPP2-1F	CAGTTTTGAGCACACCCACG	205
qBnPP2-1R	CCTAACTTCGGTCGCAGGT	
qBnPP2-2F	TGGTGGGTGGAAGTATCAGGA	187
qBnPP2-2R	TGTGGCCGTTGGATGTTGA	
qBnPP2-3F	GCAGGGAAGTACAGCGTCTT	220
qBnPP2-3R	GATTCACGACCGCAAAGCTC	
qBnPP2-4F	GGGCTTAGCAATCACTGGGA	228
qBnPP2-4R	TGGATGTTCTCGGTGTTGCA	
qBnPP2-5F	ATCCGCTTCTCATCGATGGT	176
qBnPP2-5R	TGTTCTCAGCTCAACCACCT	
qBnPP2-6F	GCTGGTTTGAAATCCGTGGC	238
qBnPP2-6R	CGCAAATGTAGCCCAAGACG	
qBnPP2-7F	GTCTCGGCCAACTAACTGCA	217
qBnPP2-7R	CTAGCCACCACACGTACGAG	
qBnPP2-8F	CTTGGACCCGGAGAGCTATC	193
qBnPP2-8R	CGACGTAATGTGTGTATGGGG	
qBnPP2-9F	TGTCAACCCGGTGAATGTGT	199
qBnPP2-9R	AAGCCCTCTCTTCCATGTGC	
qBnPP2-10F	CGAGTTTTCACCACAAGAGCA	220
qBnPP2-10R	TCGAAGAAGCTCTGCCACC	
qBnPP2-11F	CAGCGGAAGAATGGGGCAAT	199
qBnPP2-11R	TCGCCCATCTCAACCTCCAT	
qBnPP2-12F	TTGTGTGGGGTAAGGACAGC	206
qBnPP2-12R	GTAAACTTGGCAGCCGTTCC	
qBnPP2-13F	AGACAGTGGTTGGGTTAGCG	232
qBnPP2-13R	AATCACAGAAGACTTTGCGCC	
qBnPP2-14F	GCTCACTCAGGGCCTAACAA	162
qBnPP2-14R	CCAACAACAACCGAGGCAAA	
qBnPP2-15F	GGCTAAGCTTCACAATGGCG	183
qBnPP2-15R	GCCACTTCCACTCCCTTCTC	

Table S5. The primers for constructing binary expression vector

Name	Sequence (5'-3')	Length for product (bp)
pBnPP2-15-2086	CAGAGGCTATTCAAGTTTGCGG<br>ATTTGACCCTTTGCTGGGCTTG	2 086
pBnPP2-15-1455	GTGCTGCTTACGGTTTCCATG<br>ATTTGACCCTTTGCTGGGCTTG	1 455
pBnPP2-15-1039	TGCACATTATCATGTAATTAA<br>ATTTGACCCTTTGCTGGGCTTG	1 039
pBnPP2-15-579	GTAAGTCTCGATCATTACATAC<br>ATTTGACCCTTTGCTGGGCTTG	579
GFP-BnPP2-1	ATGGGCGCGTCGCTGTCGA<br>CTTCAAAACCCCTCTCCGTT	894
GFP-BnPP2-2	ATGGGGACAATTTTGTCTTGTG<br>TCTTAATAATAGGTTTTTCCTAAA	891
GFP-BnPP2-3	ATGGGAGCCAATGCGTCGTC<br>CAGCAATTCGGCCTCTTTCTCT	852
GFP-BnPP2-4	ATGGGTTCTGGTTTCTCTCTCT<br>AAAGTGCATTAACCTCCCTCTA	876
GFP-BnPP2-5	ATGTTACCAGAAGACTGTGT<br>GTTTTTAGGCCTCACTTCAAT	840
GFP-BnPP2-6	ATGAGAAAAATATTGGGGACGG<br>AGCCTTTTTGGGTCTGATTT	957
GFP-BnPP2-7	ATGGAGATGAGCAATATACCAG<br>TGCAACACCTTTAGGCCTAAG	786
GFP-BnPP2-8	ATGGAAGAAAAGGGACTAATGG<br>TGCAATCTCCTTGGGGCG	822
GFP-BnPP2-9	ATGGGAACAGGGTGGTCAC<br>ATCTTTTGCTGGCTGTATTTTG	813
GFP-BnPP2-10	ATGATAGAAGCAAAGCCTCCT<br>CATCTTTGGCTGTATACTGAC	678
GFP-BnPP2-11	ATGGAGATGAGGAATTTACCAG<br>TGTTCCAGCTTTAGGCCTA	894
GFP-BnPP2-12	ATGGCTGCAAGCAAAGCTCATC<br>ACCCGATTTAGTGACAACTGCG	531
GFP-BnPP2-13	ATGGAGCTTGGGATCGTCAAA<br>AGCAGGCAAGAGAACTCTAGTC	1173
GFP-BnPP2-14	ATGGACTTGCAATTGGTGAAAA<br>AGCGGGCATCAACAGCCA	1323
GFP-BnPP2-15	ATGGGGATTAGTGGATCAAA<br>TATCTTTGGCTGTATTTTGAC	705

* Two sequences of homologous sequences were added before the primer sequence of the cloned promoter respectively, "GACCATGATTACGCCAAGCTT" for the forward primers and "GGACTGACCACCCGGGGATCC" for the reverse primers. Two sequences of homologous sequences were added before the primer sequence of the *GFP-BnPP2s* respectively, "GCTGCGGCAGCGGCCGAATTC" for the forward primers and "TTATCTAGATCCGGTGGATCC" for the reverse primers. The red font indicates the endonuclease cleavage site.

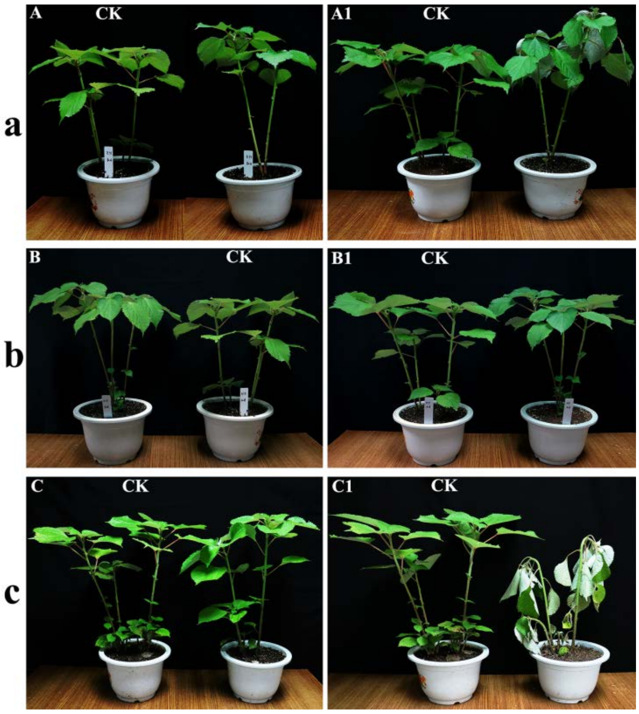

Fig. S1. The diagram of low-temperature treatment (LT), high-temperature treatment (HT) and drought stress (DS). “a” represented LT, “b” represented HT and “c” represented DS. A, B and C indicated the status before the test. A1, B1 and C1 indicated the status after the test. CK indicated control.

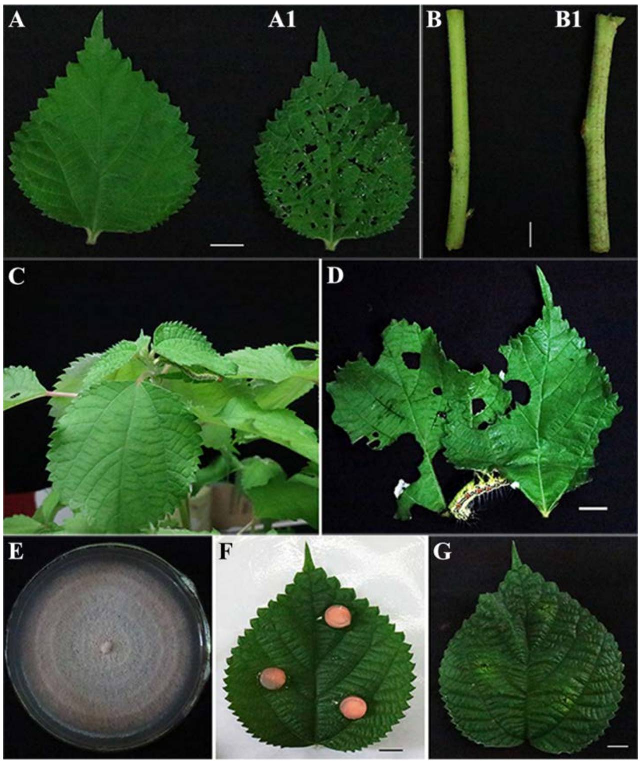

Fig. S2. The diagram of mechanical wounding treatment (MW), insect-feeding treatment (IF) and fungi infection treatment (FI). A and B indicated the status of leaves and bark before the test and A1 and B1 indicated the status after the test. C and D represented insect-feeding treatment. E was the the *colletotrichum gloeosporioides* strain isolated from the ramie leaves, F and G represented fungi infection treatment. Bar = 1cm.

>The promoter region of *BnPP2-15*, 2086 bp

```
−2 086 CAGAGGCTAT TCAAGTTTGC GGTGCATATG TATTAATGGT GGATCAAGTA GCAACTAATT
−2 026 GGTCTTTCAT TTGGTTTTTC GAGATATTTT CATTTTGTCA TTCCACACGT TTTAATTACA
−1 966 GTATATTTTT TCATCCATAT TTTATTATTT ATTTGATCGT GCAATTAATT TCATTTTTTA
−1 906 TATATATACT TTCATTTTTC TTTATGTTAT TTTCTCCTTT TTAAGTTACT TTATTTTTCC
−1 846 ATGTCATTCA ATTATATATA TTTAATTGGA CTAGTTAACT AGATAATTGT GTCTTTTATG
−1 786 AGCACGTTTA TTCATTAATT TCATTTATGA TATGATTTTC TGACGTATTG ATGTATTTAA
−1 726 TCATATTGTT ATTACATAAT TATTTTTTTT ACTTTTTATA TATATATTGT TATTGTTTAT
−1 666 GTTATAATAT TGTTATTTAA TCATATTTTT ATTATATTTA TCATATTGTT ATGGTTTATC
−1 606 ACATTGTTTC TTTATTTATC CAAATCTCTA CTTTTGACTA GCATTATTTA TTTAATTTTA
−1 546 ATTTTAATTT TAATTTTTAA TTATAAGCAA TTAATGACTT CACATTCTTT GTCGTTTTTA
−1 486 CACTTCCGTC GTTTTTTTTT TTTTTTTTAT TGTGCTGCTT ACGGTTTCCA TGTCATCTAT
−1 426 GTTTTATGTC GTTTTTCGGT TTTACGTCGT TTTTTGTGAA GCTGTCGTTC TTCGAATGTC
−1 366 GTTTTCATGT TTTGTCGTTT TCCAAGATTG ATGTCGTTTT CTACAAATAT CGTTTTACCG
−1 306 CCAAAACAGT GTCGTTTTTT TTTTTCTGCA AAGTCCCACT CTCACAAATT AACACCAAAC
−1 246 AAACACCAGG TACACAATAA AATAGCATTT CCAGCTCACA GACCCTCGTA ACTAATTTGG
−1 186 TATTCTTCTC CAAGAAAAAT GTCTTGCGTT ACGAAAAAAA AAAAAACCTA TTTGATATTT
−1 126 GGTCTTTTTT TTGCTTTATG TGTTAATGGT CAATGTTTTT AAAAATTAAA TTATTAGCTA
−1 066 TTTAATACAT AAGATAACTA AGATATATGC ACATTATCAT GTAATTAAAA AATTACTAAT
−1 006 TTTTACTATT TTTCTAAAAC ATAATTTAAA AAATAATTAT TTACTCAATT ATTTAAATAA
  −946 TAAAATTGAA AAAAGAAATT AAAAAAATCA GATGAGTCAT GACTCAGTCA TGACCTTTCT
  −886 ACTCTCGTAA TTTTCGGTCA AATATTATAG ATCTCGACCT CAACACCAAG TCAACGACGC
  −826 GTTTATTTCA TTTTATATGT CATGTCTATA TTTACTATTT TGACACTAAT ATGTCCTTTC
  −766 CAAGAAGAAG CTTCTGCGAC TAGAATAACC AAAGCAATTT CCATGAAAAT TCATCTAAAA
  −706 ATTCAAACAA ACTAACAAAA CAAAAATCAA CATTCACCAC CATAAATATA TTCACTCTCT
  −646 TCAATATTCC CAATTAGAAG TTATAATCAT CTACCTCAAC TGATAATATT GTCATCAACA
  −586 AGCGTCTGTA AGTCTCGATC ATTACATACC AAAACTTTGT TTTTACATAC TACTTTTCAT
  −526 GAATTTGTTT ATCTTTCTCT GCACTTTTTT TTTTTTTTTG CACTTGCTAG TATTAGCTTT
  −466 TACTCGAATT TCTCGGATCA TGTTCTTGGA TATATTTGCT ATGGACCTGA AATTTTGGTC
  −406 CTTATTTTGA AATATTTAGT TTGTTGAAAA GGTCCACTCT AACAGAAATG TCTTGTTTGA
  −346 GAGATTCAAT TTCGATTACA TTCAGATAAT TTTAGCCGAA AGAACATACA AACTTCAATG
  −286 GCCAAAATCA AAAGCTATAA ACAGAGTACT CTGTCATCAT TCTTGAATTA TGAGAGTTTT
  −226 AGCCTGTGCT TGTTTTTTCA CATATTTGAA CATAACTATC ATCATATCAT ATCAAATGTA
  −166 TGTGTACCTG AATTGTCATA TCACATAGTA AGACCGAAAG GGCATACGTT TTATTATGTG
  −106 CCAAAATTTG GTTAATTTCT CGACACGTGT GTGATGATGC GTCAATAATG TATATGTTGT
  − 46 TTTCATGTTT TTGTAGCGCG AGTACAAGCC CAGCAAAGGG TCAAATATG
```

Fig. S3. The promoter sequence of *BnPP2-15*. The initiation codon marked with yellow color was defined as +1. The left numbers indicated the length of the promoter.

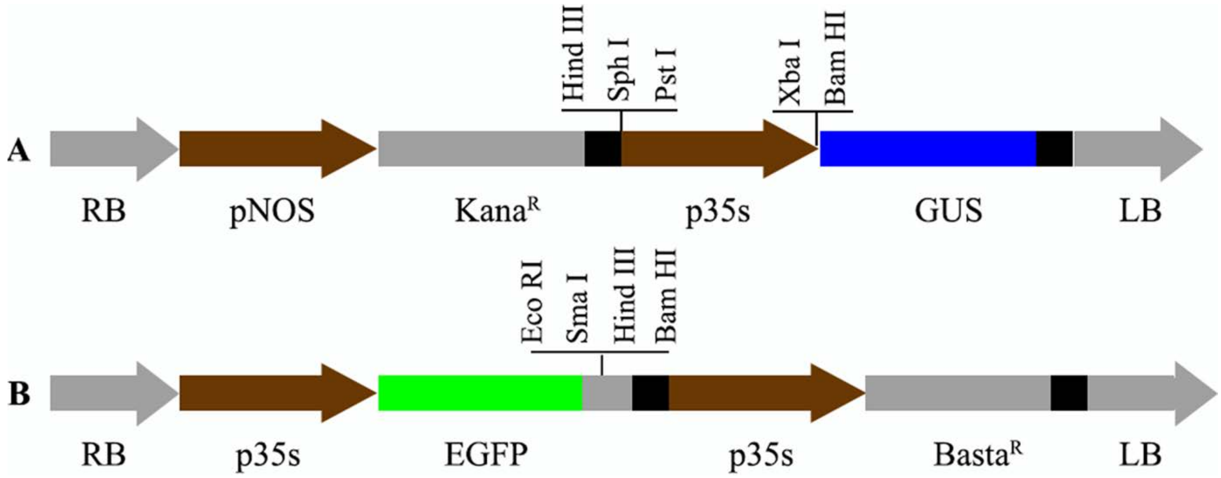

Fig. S4. T-DNA regions of binary expression plasmid pBI121and pEGAD. A represented pBI121 and B represented pEGAD. The relevant restriction sites were labeled on this figure.
